# The *Mycoplasma pneumoniae* HapE alters the cytokine profile and growth of human bronchial epithelial cells

**DOI:** 10.1042/BSR20182201

**Published:** 2019-01-18

**Authors:** Shaoli Li, Guanhua Xue, Hanqing Zhao, Yanling Feng, Chao Yan, Jinghua Cui, Hongmei Sun

**Affiliations:** Department of Bacteriology, Capital Institute of Pediatrics, Beijing 100020, China

**Keywords:** anti-inflammatory factors, cell cycle, Cytotoxicity, HapE, Mycoplasma pneumonia

## Abstract

*Mycoplasma pneumoniae* is one of the most common pathogenic causes of community-acquired pneumonia. Hydrogen sulfide, alanine, and pyruvate producing enzyme (HapE) is a recently discovered *M. pneumoniae* virulence factor that can produce H_2_S to promote erythrocyte lysis. However, other cytotoxic effects of HapE have not been explored. The present study examined the effects of this enzyme on normal human bronchial epithelial (NHBE) cells, in an attempt to identify additional mechanisms of *M. pneumoniae* pathogenesis. Recombinant HapE was purified for use in downstream assays. MTT and colony formation assays were conducted to determine the effects of HapE on cell viability and growth, while flow cytometry was used to examine changes in cell proliferation and cell cycle function. ELISA was performed to examine changes in the cytokine profile of HapE-treated cells. HapE treatment arrested NHBE cells in S phase and inhibited cell proliferation in a concentration-dependent manner. The anti-inflammatory factors interleukin (IL)-4 and IL-6 were significantly enhanced following HapE treatment. Increased secretion of pro-inflammatory factors was not observed. The effects of HapE on the respiratory epithelium may have an impact on the efficiency of host immune surveillance and pathogen elimination, and contribute to the pathogenesis of *M. pneumoniae*.

## Introduction

*Mycoplasma pneumoniae* is a prevalent pathogen in respiratory tract infections in children [1]. It causes up to 40% of community-acquired pneumonia (CAP) cases in children and as much as 18% of cases requiring hospitalization [2]. Global outbreaks of *M. pneumoniae* occur every 3–7 years. The clinical symptoms of *M. pneumoniae* infection are diverse [3]. Individuals with a mild infection can be free of symptoms. In addition to respiratory symptoms and general malaise or fever, more severe infections can cause multiple systemic extrapulmonary complications involving multiple organs and tissues [4,5].

*M. pneumoniae* infection seems to primarily involve pathogen adhesion to the respiratory epithelium, followed by direct cell invasion [6]. Oxidative stress caused by toxic oxygen molecules such as hydrogen peroxide and superoxide radicals, as well as the Community-Acquired Respiratory Distress Syndrome (CARDS) toxin, also play important roles in the pathogenesis of *M. pneumoniae*, causing extensive vacuolar degeneration and cell death in the respiratory epithelium [7–10]. It has been suggested that the chronic activation of innate immune cells during long-term *M. pneumoniae* infection may be an important factor contributing to clinical complications and the immune-evasion of the pathogen. However, the mechanism by which *M. pneumoniae* evades surveillance and pathogenic clearance by the host immune system remains poorly understood.

The hydrogen sulfide, alanine, and pyruvate producing enzyme (HapE) of *M. pneumoniae* was first characterized by Grosshennig et al. in 2016 [1,1]. In their study, the authors demonstrated that HapE can degrade cysteine ​to form H_2_S, which causes erythrocyte lysis and destruction. They therefore suggest that this enzyme is a novel potential virulence factor of *M. pneumoniae*. However, additional properties of HapE that may contribute to *M. pneumoniae* virulence have not been reported. Similarly to NO and CO, H_2_S represents a biologically active endogenous gaseous signalling molecule for humans [12–14]. It can pass through various biofilms freely. Although H_2_S is considered to be a toxic gas, recent studies have implicated endogenous H_2_S in a number of physiological and pathological processes, including haemoglobin modification, inflammation, and oxidative stress [15]. In addition, H_2_S is also utilized by pathogenic microorganisms during host infection [16,17]. During infection, pathogen-derived H_2_S can inhibit lymphocyte proliferation by affecting the synthesis of interleukin-2 (IL-2). Furthermore, H_2_S has been shown to induce phagocytes to secrete pro-inflammatory factors [18]. This up-regulation in the expression of various inflammatory mediators and cytokines can induce or aggravate inflammatory reactions, leading to tissue damage. This phenomenon has been observed for a number of pathogenic microorganisms including *Streptococcus anginosus and Paramyxovirus* [19–22]. However, other than its effects on haemolysis, the significance of HapE-dependent H_2_S production in *M. pneumoniae* infection remains to be established. Consequently, in the present study, we examined additional possible roles for HapE in the pathogenesis of *M. pneumoniae* in an attempt to provide further insight into the mechanisms driving immune damage in this disease.

## Materials and methods

### Expression and purification of recombinant proteins

The expression and purification of recombinant HapE was carried out according to the method of Grosshennig et al. [11]. Briefly, the *mpn487* gene (encoding HapE) of *M. pneumoniae* M129 (ATCC29342) was amplified by PCR, and then expressed in *Escherichia coli* Arctic-Express (Zoonbio Biotechnology Co., Ltd., China) using the pET-28a vector. Protein expression was then induced with 0.5 mM isopropyl-D-1-thiogalactopyranoside (IPTG) at 37°C for 4 h with continuous shaking at 220 rpm. The fusion protein was purified on a nickel-nitrilotriacetic acid (Ni-NTA) column according to the manufacturer’s instructions (Thermo Fisher Scientific, U.S.A.). The purity of the HapE protein was evaluated by SDS-PAGE and Western blotting. Recombinant proteins were stored at −80°C until required.

### Cell culture

The normal human bronchial epithelial (NHBE) cell line was purchased from Beijing BeNa Biotechnology Co., Ltd. Cells and were cultured at 37°C in a humidified 5% CO_2_ atmosphere in Dulbecco’s modified Eagle’s medium (DMEM) containing 10% foetal bovine serum (Thermo Fisher Scientific, U.S.A.). Cells were passaged 1:3 every other day and, upon recovery, were passaged for at least three generations before use in subsequent experiments.

### Cell proliferation and viability analysis

Cell proliferation and viability was analysed using the Vybrant MTT Cell Viability Assay (Thermo Fisher Scientific, U.S.A.) according to the manufacturer’s instructions. NHBE cells were treated with HapE (0, 0.024, 0.12, 0.3, 0.6, 3, and 15 µg/ml and the cell transfection with overexpressed vector plasmid containing the HapE gene) for 24, 48, and 72 h and then cultured with MTT solution (5 mg/ml) for an additional 4 h. The supernatant was then aspirated before the addition of 150 µl of DMSO to each well. The microtiter plate was then placed on a shaker for 10 min to facilitate the solubilisation of the formazan dye generated by viable cells. Absorbance was determined spectrophotometrically at 570 nm (Thermo Fisher Scientific, Inc., Cleveland, U.S.A.) and results were evaluated from three independent experiments.

### Colony formation assays

NHBE cells in logarithmic growth phase were dissociated into a single cell suspension with trypsin-EDTA solution, seeded into six-well plates at a density of 200 cells/well, and then cultured at 37°C for approximately 14 days. During culture, cells were treated with different concentrations of HapE (0, 0.6, 3, and 15 µg/ml). The culture medium was renewed periodically along with fresh HapE to prevent over-acidification of the medium during the course of culture. When clones could be identified macroscopically, cultures were fixed in 4% methanol for 15 min and then stained with Giemsa solution for 2 min. The number of colonies containing ≥50 cells was determined under a microscope and the efficiency of colony formation then calculated using the equation: plate clone formation efficiency (%) = (number of colonies/number of cells seeded) × 100. All tests were repeated three times.

### Cell cycle analysis

Flow cytometry in conjunction with PI staining of total cellular DNA was used to study the effects of HapE treatment on cell cycle progression. Briefly, cells were treated with various concentrations of HapE for 48 h before fixation in 75% ethanol at −20°C overnight. After washing twice with PBS, cells were incubated in PBS containing 20 µg/ml RNase at 37°C for 30 min. The cells were then stained with 0.5 mg/ml PI for 30 min in the dark at 37°C. Gating and voltage were carefully set to exclude cell clumps and debris from the analysis during flow cytometry.

## ELISA

ELISA was used to evaluate IL-2, IL-4, IL-6, IL-12, IFN-γ, and TNF-α protein levels in the culture supernatants of NHBE cells treated for 48 h with different concentrations of HapE. ELISA was performed according to the manufacturer’s instructions (R&D Systems, Minneapolis, MN, U.S.A.). All samples were run in triplicate. The optical density of samples was measured at 450 nm and protein concentrations subsequently calculated from standard curves.

### Statistical analysis

All data are expressed as means ± SD. Statistical analysis was conducted using SPSS 16.0 software (SPSS Inc., Chicago, IL, U.S.A.). Differences between experimental groups were assessed by Student’s *t*-test. A *P* value < 0.05 was considered statistically significant: *P<0.05, **P<0.01, ***P<0.001, and ****P<0.0001.

## Results

### Expression and purification of recombinant HapE

**Figure 1:**
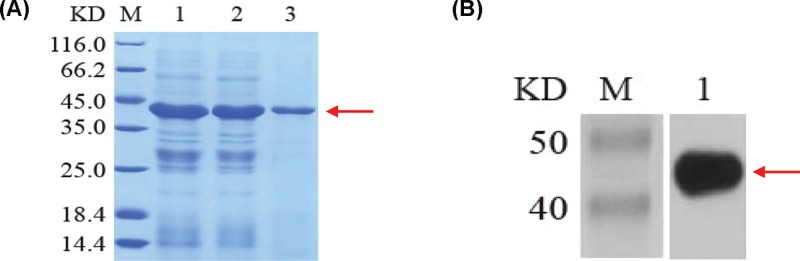
Expression, purificaton and identification of HapE protein (**A**) Expression and purification of recombinant proteins. M: molecular weight marker; lane 1: protein after induction; lane 2: elution proteins; lane 3: purified protein. (**B**). Western blotting analysis of the purified fusion protein. M: molecular weight marker; lane 1: purified protein.

HapE is an inclusion body protein and was predominantly found in the sample pellet after bacterial lysis. The protein was isolated from inclusion bodies by performing a denaturation–renaturation cycle followed by purification by Ni-NTA affinity chromatography (Figure 1A). Purified proteins were identified by Western blotting using an anti-His-tag antibody (Figure 1B).

### HapE inhibits cell viability

**Figure 2:**
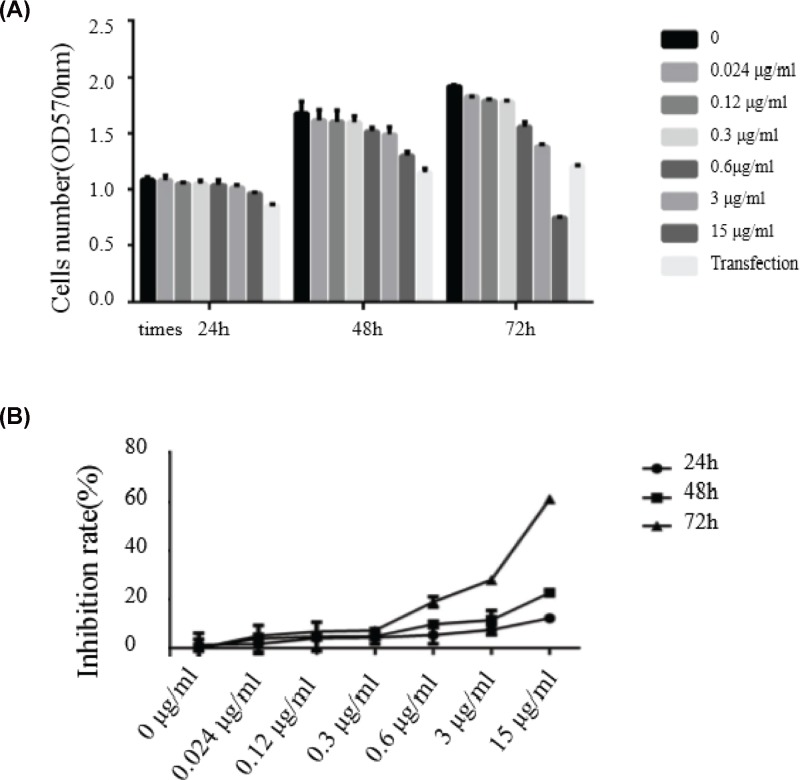
Suppression of NHBE cell viability by HapE (**A**) Cell viability, as determined by MTT assay, was measured at the indicated time points and for the indicated concentrations of recombinant HapE. One experimental group was transfected with a HapE expression plasmid for comparison. (**B**) The time-dependent inhibitory effects of increasing concentrations of HapE on cell viability, as determined by MTT assay.

NHBE cells were treated with increasing concentrations of HapE to examine its effects on cell viability. To determine the effects of intracellular HapE on viability, an additional experimental group was included where cells were transfected with a HapE expression plasmid. MTT assays revealed that HapE treatment had a dose-dependent suppressive effect on cell viability after 24 h, and that the effects of direct administration of recombinant protein were greater than those observed for transfection of cells with a HapE expression plasmid. The greatest suppression of cell viability was observed after 72 h and for the highest concentration of recombinant protein used (15 µg/ml; Figure 2).

### HapE suppresses NHBE cell colony formation

**Figure 3:**
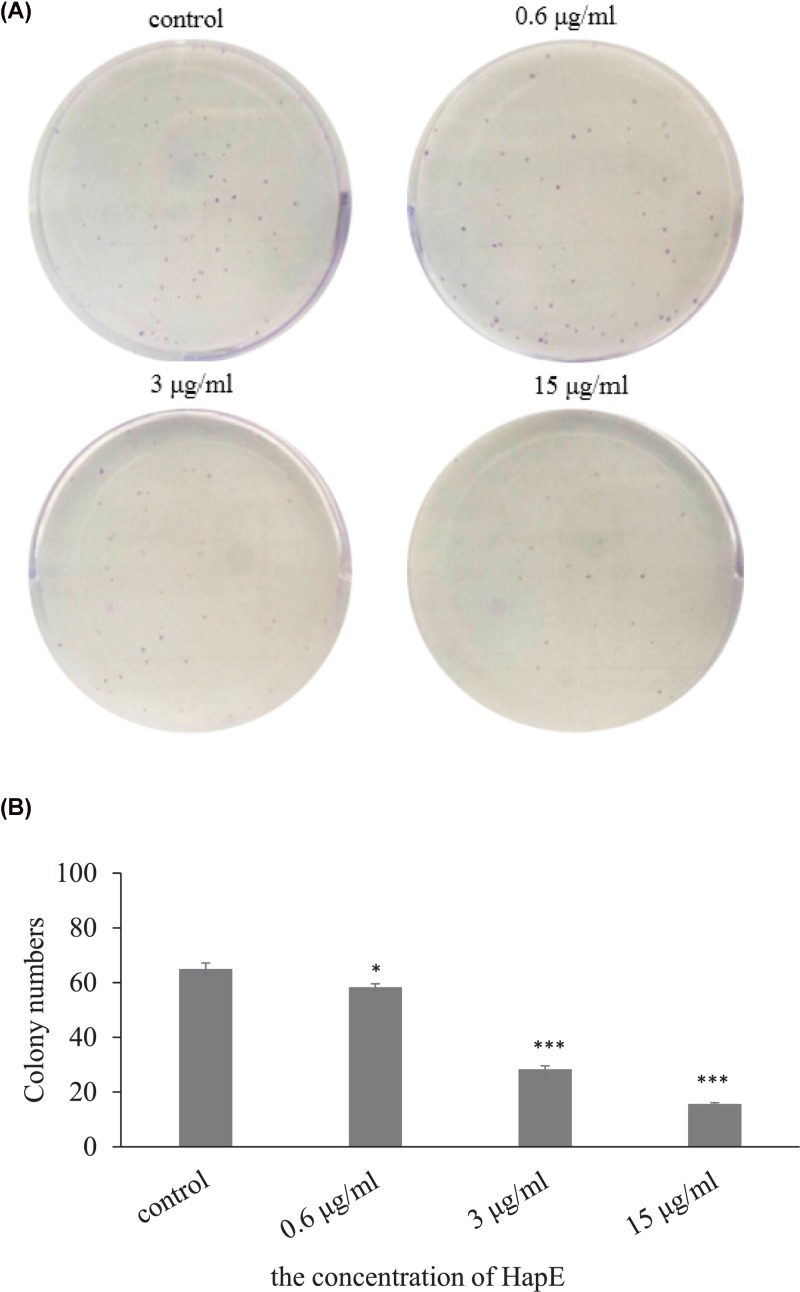
HapE suppresses NHBE cell colony formation *in vitro* (**A**). NHBE cell colonies were photographed after 14 days in culture in the indicated concentrations of recombinant HapE. (**B**). Bar graph summarizing colony numbers observed at day 14 of culture for the indicated treatment groups. Data are presented as the mean ± SD. Bar graphs summarize data from *n*=3 independent experiments. The Student’s *t-*test was used to determine the significance of differences between treatment groups (*P<0.05, ***P<0.001 compared with control group).

Next, colony formation assays were conducted to evaluate the effects of HapE on NHBE cell growth. As shown in Figure 3, fewer colonies were observed in the HapE treatment groups, when compared with the untreated control group. Moreover, HapE demonstrated a dose-dependent inhibitory effect on colony formation, with 15 µg/ml protein having the greatest suppressive effect on growth.

### HapE induces S phase arrest

**Figure 4:**
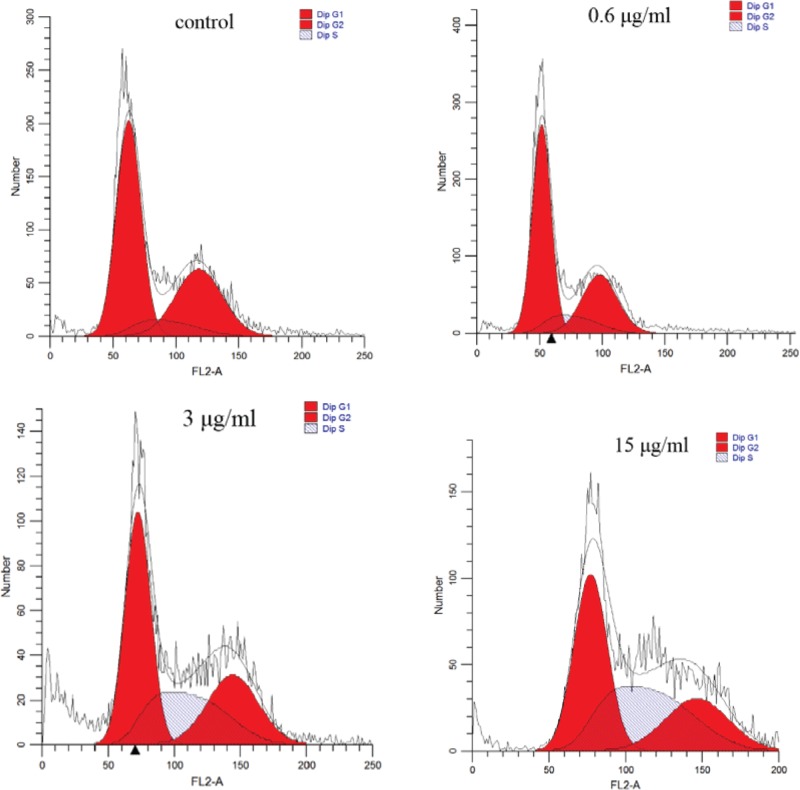
HapE promotes S phase arrest in NHBE cells

Flow cytometry was next employed to investigate the effects of HapE on the cell cycle. The flow cytometry analysis revealed that the number of NHBE cells in S phase in the 3 and 15 µg/ml HapE treatment groups was significantly increased, when compared with the cells of the 0.6 µg/ml HapE and control groups (Figure 4 and Table 1).

**Table 1:** Effects of HapE on the cell cycle distribution of NHBE cells, as quantified by flow cytometry

Group	G1 (%)	G2 (%)	S (%)
Control	56.74	33.23	10.02
0.6 μg/ml	57.49	30.20	12.31
3 μg/ml	44.52	26.15	29.33
15 μg/ml	39.98	22.18	37.83

Effects of HapE on the secretion of IL-2, IL-4, IL-6, IL-12, IFN-γ, and TNF-α by NHBE cells

**Figure 5:**
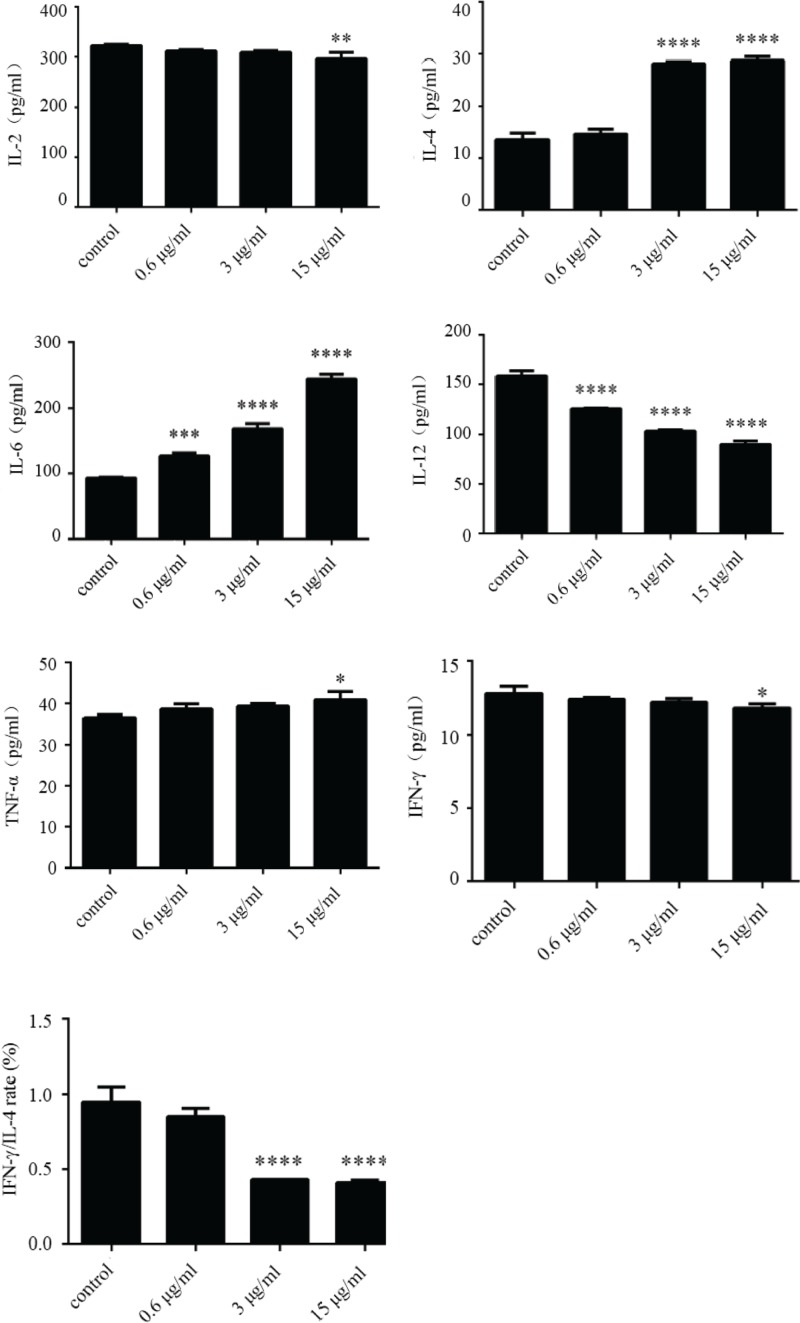
The effects of HapE treatment on the secretion of various inflammatory cytokines by NHBE cells, as measured by ELISA Data are presented as the mean ± S.D. Bar graphs summarize data from *n*=3 independent experiments. The Student’s *t-*test was used to determine the significance of differences between treatment groups (*P<0.05, **P<0.01, ***P<0.001, and ****P<0.0001 compared with control group).

The concentration of the inflammatory cytokines IL-2, IL-4, IL-6, IL-12, IFN-γ, and TNF-α in the culture supernatants of NHBE cells treated with different concentrations of recombinant HapE was measured by ELISA (Figure 5). The results showed that HapE treatment significantly inhibited the expression of the pro-inflammatory cytokine IL-12, had little effect on the expression of IL-2, IFN-γ, and TNF-α, and significantly increased the expression of the anti-inflammatory cytokines IL-6 and IL-4 (>3 µg/ml). The observed alteration in the ratio of IFN-γ to IL-4 expression is shown in Figure 5. Levels of the cytokine IL-4 (an inducer of Th2 cell differentiation) in the culture supernatants of the 3 and 15 µg/ml treatment groups were significantly elevated, when compared with the control group (****P<0.0001). Only the highest concentration of HapE tested (15 µg/ml) resulted in a significant reduction in IFN-γ levels (*P<0.05). The ratio of IFN-γ to IL-4 in the supernatants of the 3 and 15 µg/ml HapE treatment groups was significantly reduced, when compared with the control group (****P<0.0001).

## Discussion

*M. pneumoniae* is one of the most common pathogen associated with CAP in children and young adults [6]. In addition to causing respiratory diseases, *M. pneumoniae* can also cause multiple systemic complications that can have serious consequences, affecting the patient’s physical and mental health [23–25]. CAP frequently presents as a mixed infection, with *M. pneumoniae* co-existing with, and aggravating the clinical symptoms caused by other pathogens [26]. Patients who have suffered *M. pneumoniae* infection often experience recurrent and prolonged disease, and significantly impaired immunity [23,27]. Therefore, it is important to understand the molecular mechanisms driving the pathogenesis of *M. pneumoniae*.

It is now largely accepted that, in addition to directly causing host cell damage, *M. pneumoniae* infection also suppresses host immune function and disrupts humoral immunity [23,28]. The host inflammatory response is necessary to eliminate pathogenic microorganisms. The innate immune system, including the complement system, is the host’s first line of defence against foreign pathogens and is therefore crucial in determining the outcome of a pathogen–host confrontation. A decrease in the secretion of inflammatory factors leads to a decrease in both the inflammatory response and the efficiency of pathogen clearance [29]. Therefore, inhibition of the production of inflammatory mediators facilitates the immune evasion and sustained survival of pathogenic microorganisms. Previous studies have shown that a Th1/Th2 cytokine profile plays an important role in the immunological response [30,31]. Studies have shown that *M. pneumoniae* pneumonia can result in the down-regulation of Th1 cytokines and/or the up-regulation of Th2 cytokines, thereby shifting the Th1/Th2 cell balance towards a Th2-dominant phenotype. However, questions remain as to how *M. pneumoniae* causes this imbalance in Th1/Th2 cytokines, and which specific virulence factors are involved in this process.

The *M. pneumoniae* HapE protein was first discovered in 2016 by Grosshennig et al. [11], who showed that this enzyme catalyses the decomposition of cysteine to H_2_S. They also demonstrated that HapE-dependent H_2_S generation can cause erythrocyte lysis, suggesting that HapE is a virulence factor in *M. pneumoniae* pneumonia. However, other than its effects on erythrocyte lysis, the role of HapE in the virulence of *M. pneumoniae* remains largely unexplored. To study the role of HapE in the virulence of *M. pneumoniae*, we studied the effects of this protein *in vitro* using NHBE cells, which provide a useful model for the respiratory epithelium. Our study demonstrated that HapE had cytostatic or even cytotoxic effects when expressed at high levels (>3 μg/ml), and that HapE interfered with cell cycle progression. These effects were dependent upon both the protein concentration and the duration of action. These findings reveal that HapE also has a significant influence on cell proliferation and survival. Therefore, we speculate that if the amount of bacteria infected by *M. pneumoniae in vivo* is high, the level of HapE toxin in the body is also high, the degree of damage to the cells will be heavy, and the impact on the cell cycle will be large. This phenomenon may be beneficial for the survival and reproduction of *M. pneumoniae* within cells, which impedes pathogen clearance and prolongs the duration of infection.

In this study, ELISA was used to examine changes in the concentration of IL-2, IL-4, IL-6, IL-12, IFN-γ, and TNF-α in the supernatants of HapE-treated NHBE cells. Of these cytokines, IL-2, IL-12, IFN-γ, and TNF-α are secreted by Th1 cells and can promote cell-mediated immune responses to intracellular microbial pathogens. IL-4 and IL-6, however, are mainly secreted by Th2 cells and can promote humoral immune responses to extracellular microbial pathogens and also the secretion of protective antibodies [32]. Our study has shown that, in addition to TNF-α, the other Th1 cytokines investigated (IL-2, IL-12, and IFN-γ) decreased, while the Th2 cytokines (IL-4 and IL-6) increased. The Th1 cytokines IL-2, IL-12, and IFN-γ, promote inflammation. However, in the present study, the levels of these cytokines were either unchanged or reduced in the supernatants of HapE-treated cells. This suggests that HapE is not associated with the secretion of pro-inflammatory factors. However, IL-4 is an anti-inflammatory cytokine that can inhibit macrophage activation, and may therefore prevent *M. pneumoniae* clearance, thereby increasing the chance of chronic infection. IL-6 is an important cytokine that has dual functions in the process of inflammation [32]. Our study showed that the level of IL-6 secreted by NHBE cells increased significantly with increasing concentrations of HapE. This observation suggests that IL-6, which is an important Th2 cytokine, may have an anti-inflammatory role in the pathogenesis of *M. pneumoniae* pneumonia, and that HapE, through its effects on IL-6 secretion, may contribute to the Th1/Th2 imbalance associated with this disease. Our finding that HapE treatment resulted in a significant decrease in the IFN-γ to IL-4 ratio suggests that this protein primarily drives a Th2-type cell response. When taken together, therefore, the findings from our study provide a possible basis for the mechanism of immune escape during *M. pneumoniae* infection.

However, there are some limitations in our study, like there is no *in vivo* data or *ex vivo* data to translate these *in vitro* findings into a physiologically relevant context, and did not consider the use of primary human tissue or an animal model, and no verification of whether other bacteria express this enzyme, etc. Therefore, In the future, more research is needed to further study the function of this protein.

## Conclusion

HapE inhibits the proliferation of NHBE cells, arresting cells in S phase. These effects are dependent upon the protein concentration and the duration of action. HapE and its metabolites act on host cells to reduce the secretion of inflammatory factors and increase the release of anti-inflammatory mediators, thereby facilitating *M. pneumoniae* evasion of the host immune system. These findings, therefore, provide a possible basis for the sustained survival and replication of *M. pneumoniae*, which can cause chronic infection and the prolongation of symptoms associated with this disease.

## Perspectives

- The HapE is a recently discovered *M. pneumoniae* virulence factor that can produce H_2_S to promote erythrocyte lysis. However, other cytotoxic effects of HapE have not been explored.
- HapE treatment arrested the respiratory epithelium in S phase and inhibited cell proliferation in a concentration-dependent manner. The anti-inflammatory factors were significantly enhanced following HapE treatment. Increased secretion of pro-inflammatory factors was not observed.
- The effects of HapE on the respiratory epithelium have an impact on the efficiency of host immune surveillance and pathogen elimination, and contribute to the pathogenesis of *M. pneumoniae*.

## Abbreviations

CAP: community-acquired pneumonia
HapE: hydrogen sulfide, alanine, and pyruvate producing enzyme
IL: interleukin
NHBE: normal human bronchial epithelial
Ni-NTA: nickel-nitrilotriacetic acid

## References

[1] Cilloniz C., Ewig S., Polverino E., Marcos M.A., Prina E., Sellares J. (2012) Community-acquired pneumonia in outpatients: aetiology and outcomes. Eur. Respir. J. 40, 931–938 10.1183/09031936.00168811 22267760

[2] Sondergaard M.J., Friis M.B., Hansen D.S. and Jorgensen I.M. (2018) Clinical manifestations in infants and children with Mycoplasma pneumoniae infection. PLoS One 13, e0195288 10.1371/journal.pone.0195288 29698412 PMC5919654

[3] Neamtu L., Sciuca S. and Selevestru R. Clinical peculiarities of mycoplasma infection in children with community pneumonia2016. PA1273 p

[4] Kutty P.K., Jain S., Taylor T.H., Bramley A.M., Diaz M.H., Ampofo K. (2018) Mycoplasma Pneumoniae among Children Hospitalized with Community-acquired Pneumonia. Clin. Infect. Dis., 10.1093/cid/ciy419 PMC6552676 29788037

[5] Bajantri B., Venkatram S. and Diaz-Fuentes G. (2018) Mycoplasma pneumoniae: A Potentially Severe Infection. J. Clin. Med. Res. 10, 535–544 10.14740/jocmr3421w 29904437 PMC5997415

[6] Waites K.B., Xiao L., Liu Y., Balish M.F. and Atkinson T.P. (2017) Mycoplasma pneumoniae from the Respiratory Tract and Beyond. Clin. Microbiol. Rev. 30, 747–809 10.1128/CMR.00114-16 28539503 PMC5475226

[7] Becker A., Kannan T.R., Taylor A.B., Pakhomova O.N., Zhang Y., Somarajan S.R. (2015) Structure of CARDS toxin, a unique ADP-ribosylating and vacuolating cytotoxin from Mycoplasma pneumoniae. Proc. Natl. Acad. Sci. U.S.A. 112, 5165–5170 10.1073/pnas.1420308112 25848012 PMC4413325

[8] Medina J.L., Coalson J.J., Brooks E.G., Le Saux C.J., Winter V.T., Chaparro A. (2014) Mycoplasma pneumoniae CARDS toxin exacerbates ovalbumin-induced asthma-like inflammation in BALB/c mice. PLoS One 9, e102613 10.1371/journal.pone.0102613 25058417 PMC4109942

[9] Kannan T.R., Krishnan M., Ramasamy K., Becker A., Pakhomova O.N., Hart P.J. (2014) Functional mapping of community-acquired respiratory distress syndrome (CARDS) toxin of Mycoplasma pneumoniae defines regions with ADP-ribosyltransferase, vacuolating and receptor-binding activities. Mol. Microbiol. 93, 568–581 10.1111/mmi.12680 24948331 PMC4116743

[10] Bose S., Segovia J.A., Somarajan S.R., Chang T.H., Kannan T.R. and Baseman J.B. (2014) ADP-ribosylation of NLRP3 by Mycoplasma pneumoniae CARDS toxin regulates inflammasome activity. MBio 5, 10.1128/mBio.02186-14 PMC4278538 25538194

[11] Grosshennig S., Ischebeck T., Gibhardt J., Busse J., Feussner I. and Stulke J. (2016) Hydrogen sulfide is a novel potential virulence factor of Mycoplasma pneumoniae: characterization of the unusual cysteine desulfurase/desulfhydrase HapE. Mol. Microbiol. 100, 42–54 10.1111/mmi.13300 26711628

[12] Kumar M. and Sandhir R. (2018) Hydrogen sulfide in physiological and pathological mechanisms in brain. CNS Neurol. Disord. Drug Targets, 10.2174/1871527317666180605072018 29866024

[13] Cuevasanta E., Moller M.N. and Alvarez B. (2017) Biological chemistry of hydrogen sulfide and persulfides. Arch. Biochem. Biophys. 617, 9–25 10.1016/j.abb.2016.09.018 27697462

[14] Chan S.J. and Wong P.T. (2017) Hydrogen sulfide in stroke: protective or deleterious? Neurochem. Int. 105, 1–10 28174023 10.1016/j.neuint.2016.11.015

[15] Hu X., Chi Q., Wang D., Chi X., Teng X. and Li S. (2018) Hydrogen sulfide inhalation-induced immune damage is involved in oxidative stress, inflammation, apoptosis and the Th1/Th2 imbalance in broiler bursa of Fabricius. Ecotoxicol. Environ. Saf. 164, 201–209 10.1016/j.ecoenv.2018.08.029 30118953

[16] Wareham L.K., Southam H.M. and Poole R.K. (2018) Do nitric oxide, carbon monoxide, and hydrogen sulfide really qualify as ‘gasotransmitters’ in bacteria? Biochem. Soc. Trans. 10.1042/BST20170311 30190328 PMC6195638

[17] Hotaling S., Quackenbush C.R., Bennett-Ponsford J., New D.D., Arias-Rodriguez L., Tobler M. (2018) Bacterial diversity in replicated hydrogen sulfide-rich streams. Microb. Ecol. 10.1007/s00248-018-1237-6 30105506

[18] Bazhanov N., Escaffre O., Freiberg A.N., Garofalo R.P. and Casola A. (2017) Broad-range antiviral activity of hydrogen sulfide against highly pathogenic RNA viruses. Sci. Rep. 7, 41029 10.1038/srep41029 28106111 PMC5247713

[19] Verma S., Landisch R., Quirk B., Schmainda K., Prah M., Whelan H.T. (2013) Presumed hydrogen sulfide-mediated neurotoxicity after streptococcus anginosus group meningitis. Pediatr. Infect. Dis. J. 32, 189–191 10.1097/INF.0b013e3182748fe9 23014355 PMC3548939

[20] Nakamura S., Shioya K., Hiraoka B.Y., Suzuki N., Hoshino T., Fujiwara T. (2018) Porphyromonas gingivalis hydrogen sulfide enhances methyl mercaptan-induced pathogenicity in mouse abscess formation. Microbiology 164, 529–539 10.1099/mic.0.000640 29488863

[21] Chi X.P., Ouyang X.Y. and Wang Y.X. (2014) Hydrogen sulfide synergistically upregulates Porphyromonas gingivalis lipopolysaccharide-induced expression of IL-6 and IL-8 via NF-kappaB signalling in periodontal fibroblasts. Arch. Oral. Biol. 59, 954–961 10.1016/j.archoralbio.2014.05.022 24927331

[22] Li H., Ma Y., Escaffre O., Ivanciuc T., Komaravelli N., Kelley J.P. (2015) Role of hydrogen sulfide in paramyxovirus infections. J. Virol. 89, 5557–5568 10.1128/JVI.00264-15 25740991 PMC4442521

[23] Saraya T. (2017) Mycoplasma pneumoniae infection: Basics. J. Gen. Fam. Med. 18, 118–125 10.1002/jgf2.15 29264006 PMC5689399

[24] Al Busaidi I., Al-Amin M., Ibrahim S., Balkhair A. and Gaifer Z. (2017) Multi-system manifestations of Mycoplasma pneumoniae infection in a young patient. JMM Case Rep. 4, e005117 10.1099/jmmcr.0.005117 29114398 PMC5643005

[25] Chaudhry R., Ghosh A. and Chandolia A. (2016) Pathogenesis of Mycoplasma pneumoniae: An update. Indian J. Med. Microbiol. 34, 7–16 10.4103/0255-0857.174112 26776112

[26] Zhang X., Chen Z., Gu W., Ji W., Wang Y., Hao C. (2018) Viral and bacterial co-infection in hospitalised children with refractory Mycoplasma pneumoniae pneumonia. Epidemiol. Infect. 146, 1384–1388 10.1017/S0950268818000778 29970200 PMC9133674

[27] Rogozinski L.E., Alverson B.K. and Biondi E.A. (2017) Diagnosis and treatment of Mycoplasma pneumoniae in children. Minerva Pediatr. 69, 156–160 28178776 10.23736/S0026-4946.16.04866-0

[28] Chkhaidze I. and Kapanadze N. (2017) Cytokines as the predictors of severe Mycoplasma Pneumoniae Pneumonia in children (Review). Georgian Med. News 89–95 28726662

[29] Shimizu T., Kimura Y., Kida Y., Kuwano K., Tachibana M., Hashino M. (2014) Cytadherence of Mycoplasma pneumoniae induces inflammatory responses through autophagy and toll-like receptor 4. Infect. Immun. 82, 3076–3086 10.1128/IAI.01961-14 24799628 PMC4097642

[30] Zhao J.L., Wang X. and Wang Y.S. (2016) Relationships between Th1/Th2 cytokine profiles and chest radiographic manifestations in childhood Mycoplasma pneumoniae pneumonia. Ther. Clin. Risk Manag. 12, 1683–1692 10.2147/TCRM.S121928 27956836 PMC5113916

[31] Li W., Liu Y.J., Zhao X.L., Shang S.Q., Wu L., Ye Q. (2016) Th1/Th2 Cytokine profile and its diagnostic value in Mycoplasma pneumoniae Pneumonia. Iran J. Pediatr. 26, e3807 10.5812/ijp.3807 26848377 PMC4733293

[32] Akdis M., Aab A., Altunbulakli C., Azkur K., Costa R.A., Crameri R. (2016) Interleukins (from IL-1 to IL-38), interferons, transforming growth factor beta, and TNF-alpha: Receptors, functions, and roles in diseases. J. Allergy Clin. Immunol. 138, 984–1010 10.1016/j.jaci.2016.06.033 27577879

